# Bayesian Monte Carlo Simulation Based on Systematic Review for Personalized Risk Stratification of Contralateral Lymph Node Metastasis in Oral Squamous Cell Carcinoma †

**DOI:** 10.3390/diagnostics15212668

**Published:** 2025-10-22

**Authors:** Karthik N. Rao, M. P. Sreeram, Prajwal Dange, Andres Coca Pelaz, Cesare Piazza, Remco de Bree, Fernando Lopez, Orlando Guntinas-Lichius, Luiz Paulo Kowalski, Kevin T. Robbins, Primož Strojan, Carlos Suárez, Akihiro Homma, Robert Takes, Juan Pablo Rodrigo, Marc Hamoir, Avraham Eisbruch, Francisco Civantos, Anna Luíza Damaceno Araújo, Alessandra Rinaldo, Małgorzata Wierzbicka, Alfio Ferlito

**Affiliations:** 1 Department of Head and Neck Oncology, Sri Shankara Cancer Foundation, Bangalore 560004, India; sreeram.mp@sschrc.org (M.P.S.); prajwal.dange@gmail.com (P.D.); 2 Department of Otolaryngology, Hospital Universitario Central de Asturias, ISPA, IUOPA, CIBERONC, University of Oviedo, 33003 Oviedo, Spain; acocapelaz@yahoo.es (A.C.P.); fernandolopezphd@gmail.com (F.L.); jprodrigo@uniovi.es (J.P.R.); 3 Unit of Otorhinolaryngology-Head and Neck Surgery, ASST Spedali Civili of Brescia, Department of Surgical and Medical Specialties, Radiological Sciences and Public Health, School of Medicine, University of Brescia, 25123 Brescia, Italy; ceceplaza@libero.it; 4 Department of Head and Neck Surgical Oncology, University Medical Center Utrecht, 3584 CX Utrecht, The Netherlands; r.debree@umcutrecht.nl; 5 Department of Otorhinolaryngology, Institute of Phoniatry/Pedaudiology, Jena University Hospital, 07747 Jena, Germany; orlando.guntinas@med.uni-jena.de; 6 Department of Head and Neck Surgery, University of São Paulo Medical School, São Paulo 05403-000, SP, Brazil; lp_kowalski@uol.com.br (L.P.K.); anna_luizaf5ph@hotmail.com (A.L.D.A.); 7 Department of Head and Neck Surgery, A. C. Camargo Cancer Center, São Paulo 01525-001, SP, Brazil; 8 Department of Otolaryngology Head Neck Surgery, SIU School of Medicine, Southern Illinois University, Springfield, IL 62702, USA; kthomasrobbins@gmail.com; 9 Department of Radiation Oncology, Institute of Oncology, 1000 Ljubljana, Slovenia; pstrojan@onko-i.si; 10 Universidad de Oviedo, 33011 Oviedo, Spain; csuareznieto@gmail.com; 11 Department of Otolaryngology-Head and Neck Surgery, Faculty of Medicine, Graduate School of Medicine, Hokkaido University, Sapporo 060-0808, Japan; akihomma@gmail.com; 12 Department of Otolaryngology-Head and Neck Surgery, Radboud University Medical Center, 6500 HB Nijmegen, The Netherlands; robert.takes@radboudumc.nl; 13 Department of Otorhinolaryngology-Head and Neck Surgery, St Luc University Hospital and King Albert II Cancer Institute, UC Louvain, 1200 Brussels, Belgium; marc.hamoir@saintluc.uclouvain.be; 14 Department of Radiation Oncology, University of Michigan, Ann Arbor, MI 48109, USA; eisbruch@med.umich.edu; 15 Department of Otolaryngology-Head and Neck Surgery, School of Medicine, University of Miami Miller, Miami, FL 33136, USA; fcivanto@med.miami.edu; 16 Hospital Israelita Albert Einstein, São Paulo 05652-900, SP, Brazil; 17 ENT Unit, Policlinico Città di Udine, 33100 Udine, Italy; dottalerinaldo@gmail.com; 18 Regional Specialist Hospital Wroclaw, Research & Development Centre, 51-124 Wroclaw, Poland; wierzbicka.otolaryngology@gmail.com; 19 Faculty of Medicine, Wroclaw University of Science and Technology, 50-370 Wroclaw, Poland; 20 Coordinator of the International Head and Neck Scientific Group, 35100 Padua, Italy; profalfioferlito@gmail.com

**Keywords:** oral squamous cell carcinoma, contralateral lymph node metastasis, Monte Carlo simulation, risk stratification, Bayesian modelling

## Abstract

**Background**: Contralateral lymph node metastasis (CLNM) in oral squamous cell carcinoma (OSCC) represents a major clinical challenge, in patients with a clinically contralateral node-negative neck. Individualized risk stratification is crucial to guide decisions on elective contralateral neck dissection. This study aimed to synthesize existing evidence and apply Bayesian Monte Carlo Simulation (MCS) to estimate CLNM probability across various clinic-pathological scenarios. **Methods**: A systematic search of PubMed, PubMed Central, and Embase (2000–2024) identified 26 eligible studies. Effect sizes for seven key risk factors—midline-crossing tumours, extranodal extension (ENE), ≥2 ipsilateral lymph nodes, depth of invasion (DOI) >10 mm, perineural invasion and lymphovascular invasion (PNI-LVI), poor differentiation, and floor of mouth subsite—were computed and incorporated into a Bayesian logistic model. Using the No-U-Turn Sampler (NUTS) in RStan, 100,000 virtual patient profiles were simulated to generate posterior probabilities of CLNM. **Results**: The baseline CLNM risk for lateralized tumours without additional risk factors was 4.2%. Single risk factors increased probability substantially: midline-crossing tumours (31.7%), ENE (27.4%), and ≥2 ipsilateral nodes (24.9%). Combinations of risk factors amplified the risk non-linearly: the presence of a midline-crossing tumour, ENE, and ≥2 ipsilateral nodes yielded a 76.8% CLNM probability, and the presence of all seven risk factors increased it to 93.7%. Risk tiers were classified from minimal (<20%) to very high (>50%) to guide clinical decision-making. **Conclusions**: This MCS-based model reveals that CLNM risk increases multiplicatively with the presence of various high-risk features. The simulation supports bilateral neck management in high-risk patients and observation in low-risk cases. Prospective validation is needed to integrate this model into routine clinical practice and to guide patient-specific surgical planning.

## 1. Introduction

Oral squamous cell carcinoma (OSCC) is the most common malignancy of the head and neck region, with more than 389,000 new cases reported annually worldwide, according to GLOBOCAN 2022 [1]. The cornerstone of curative management in locally advanced OSCC includes wide local excision of the primary tumour and elective neck dissection for regional control [2,3,4,5,6]. While management of the ipsilateral neck is well established, the treatment of the contralateral neck, especially in clinically node-negative (cN0) on contralateral side, remains debated due to the lack of robust evidence on survival benefit. Reported rates of contralateral lymph node metastasis (CLNM) range widely from 1% to 35%. This variability reflects heterogeneity in tumour subsite, laterality, staging, and biological behaviour [5,7].

Elective contralateral neck dissection is associated with increased operative morbidity and functional deficits, particularly shoulder dysfunction, without consistent evidence of survival benefit. Therefore, accurate preoperative risk stratification is critical to guide bilateral neck management and avoid overtreatment in low-risk patients. Multiple clinical, radiological, and pathological risk factors—including tumour proximity to or crossing of the midline, extranodal extension (ENE), depth of invasion (DOI), perineural invasion (PNI), lymphovascular invasion (LVI), and nodal burden—have been individually associated with CLNM risk [8,9,10,11]. However, the cumulative and interactive impact of these variables is often underappreciated in clinical decision-making due to the absence of integrated, probabilistic models.

Given these uncertainties, we conducted a systematic review to synthesize existing evidence and, building upon this foundation, developed a Bayesian Monte Carlo Simulation model (MCS) for risk estimation [12,13]. Unlike conventional models that offer point estimates, MCS captures uncertainty and enables personalized, quantitative predictions of contralateral spread. MCS allows direct incorporation of prior evidence and provides interpretable probability distributions, which may be more clinically meaningful than point estimates. Our goal is to offer a practical and adaptable risk stratification model that can support surgical decision-making in managing the contralateral neck in OSCC. To our knowledge, this is the first study to combine a systematic review with a Bayesian MCS framework to produce clinically interpretable, patient-specific probabilities of CLNM.

## 2. Methodology

**Figure 1:**
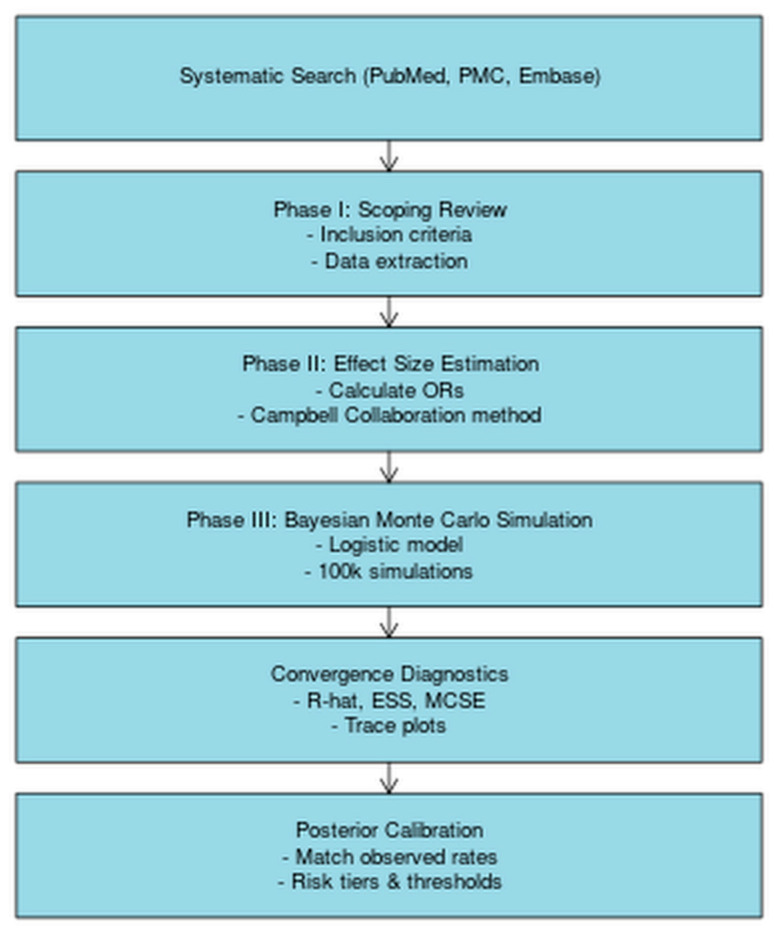
Simplified methodology flowchart of the Bayesian Monte Carlo simulation study.

To obtain a comprehensive collection of the relevant medical literature, a systematic search was conducted across PubMed, PubMed Central, and Embase databases. The search was restricted to English-language publications from the year 2000 to 2024. The study was structured into 3 sequential phases: (1) a systematic review, (2) evidence synthesis and estimation of effect sizes (odds ratios or posterior probabilities), and (3) an MCS using the derived posterior estimates (Figure 1).

The study was conducted in three sequential phases, beginning with a systematic review, followed by effect size estimation, and culminating in Bayesian simulation with convergence and calibration analysis.

### 2.1. Phase I: Systematic Review

In the first phase, we systematically identified and evaluated risk factors associated with CLNM in OSCC. A structured search strategy was implemented on PubMed, using a combination of keywords such as “oral cancer”, “squamous cell carcinoma”, “lymph node metastasis”, “contralateral neck”, “neck dissection”, “risk factors”, “sentinel node biopsy”, “tongue”, “buccal mucosa”, and “floor of mouth”. Boolean operators “AND” and “OR” were applied to refine results. The final search was completed on 15 November 2024. This project was not registered in PROSPERO.

**Figure 2:**
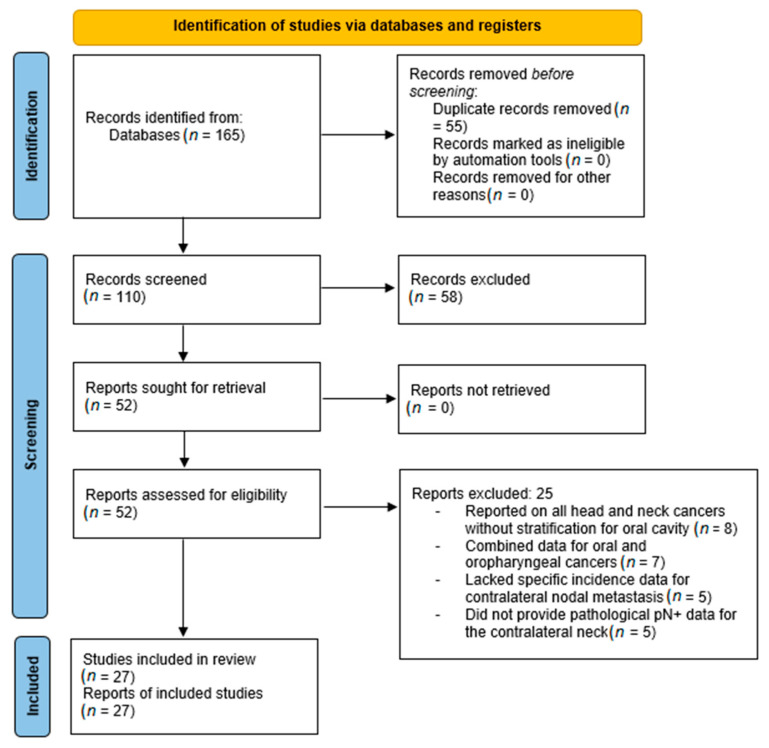
PRISMA 2020 flow diagram for study selection.

Three reviewers (KNR, SMP, and PD) independently screened the retrieved studies by evaluating article type, title, and abstract. A full-text review was conducted for eligible articles. PD and SMP finalized the study selection, with any discrepancies resolved through discussion with KNR (Figure 2).

Studies were included if they met the following criteria: they reported on patients with primary OSCC, evaluated the presence or risk of CLNM, provided risk factor-specific outcomes, and discussed surgical or surveillance strategies for managing the contralateral neck. Both retrospective and prospective studies were eligible for inclusion. After study selection, data extraction was performed independently by PD and SMP. The variables extracted included tumour lateralization (categorized as unilateral, midline-reaching, or crossing), DOI and tumour thickness, ipsilateral nodal involvement, ENE, PNI, LVI, histopathological grade, tumour subsite, clinical stage, and number of metastatic lymph nodes. Risk-of-bias assessment using the Newcastle–Ottawa Scale (NOS) for all included observational studies was performed.

### 2.2. Phase II: Effect Size and Probability Estimation

The second phase involved synthesis of the extracted data to estimate effect sizes. For each identified risk factor, the rate of CLNM was recorded across the included studies. Odds ratios (ORs) were computed using standard formulae provided by the Campbell Collaboration’s Effect Size Calculator (https://www.campbellcollaboration.org/calculator/equations, accessed on 23 July 2025), serving as a basis for quantitative modelling in the next phase.

### 2.3. Phase III: Bayesian MCS

In the final phase, a Bayesian MCS was employed to estimate the uncertainty in CLNM risk prediction, integrating synthesized evidence from the systematic review into a hierarchical modelling framework [14,15].

Informative priors were specified for each risk factor’s effect size, using log-odds-transformed ORs from the systematic review. The likelihood function assumed a Bernoulli distribution for CLNM occurrence, with individual event probabilities modelled using a logistic regression function that incorporated all identified risk factors. Covariates were sampled from their empirical frequency distributions as reported in the literature.

Markov Chain Monte Carlo (MCMC) sampling was conducted using the No-U-Turn Sampler (NUTS) algorithm, with 4 chains run for 25,000 iterations each (10,000 iterations for warm-up). Convergence was assessed using R-hat statistics and trace plots. The simulation generated 100,000 synthetic patient profiles, yielding full posterior distributions of CLNM risk across clinically relevant combinations of predictors. Model calibration was verified through posterior predictive checks comparing simulation outputs with reported incidence rates from the literature. Clinical decision thresholds were derived by analyzing the posterior probability mass across various risk strata.

Convergence was rigorously assessed using multiple diagnostics, including the Gelman–Rubin R-hat statistic, where values below 1.05 indicated acceptable convergence between and within chains. The Effective Sample Size (ESS) was examined for each parameter to confirm sufficient independent samples, while Monte Carlo Standard Error (MCSE) was evaluated relative to the posterior standard deviation to ensure simulation precision. Additionally, trace plots and density overlays were visually inspected to assess chain mixing and stationarity, and autocorrelation analysis was conducted to verify minimal dependence between successive samples. All analyses were implemented in R (version 4.3.1 for Windows).

## 3. Results

### 3.1. Phase I: Systematic Search Summary and Study Selection

A total of 165 records were identified through database searches (PubMed, PMC, and Embase). After removing 55 duplicates, 110 unique records were screened. Title and abstract screening excluded 58 records, leaving 52 reports for full-text review. No reports were excluded due to retrieval issues. Among the 52 full-text articles assessed, 25 were excluded for the following reasons: eight studies reported on all head and neck cancers without stratification for the oral cavity, seven combined data for oral and oropharyngeal cancers, five lacked specific incidence data for CLNM, and five did not provide pathological pN+ data for the contralateral neck. Ultimately, 27 studies met the inclusion criteria and were included in the qualitative synthesis (Figure 2). The quality assessment using the NOS indicated that all included studies scored between 6 and 7, reflecting moderate to good methodological quality.

### 3.2. Phase II: Systematic Review

CLNM in OSCC is strongly influenced by tumour laterality and the presence of specific high-risk pathological features. Consistent with prior evidence, lateralized lesions—defined as those located more than 1 cm from the midline—generally carry the lowest risk of CLNM. Liu et al. reported that among patients with oral tongue and floor of mouth cancers, a DOI > 10 mm and ipsilateral nodal positivity conferred a 5% contralateral failure rate in untreated neck [16]. Udovicich et al. noted a 15.2% pathological node positivity in the contralateral neck in lateralized tumours, particularly when associated with high N stage, DOI > 6 mm, PNI, and ENE [17]. Doll et al. reported that a cumulative 9.2% of treated and untreated patients developed CLNM during follow-up [10]. Habib et al. reported that only 2.9% of patients with lateralized oral cavity tumours experienced isolated CLNM (ipsilateral N0) following observation, although the risk rose to 10% in poorly differentiated tumours or those with ipsilateral nodal metastases [18]. Koo et al. observed an 11% incidence of occult CLNM (cN0 but pN+) in lateralized lesions, particularly when ipsilateral nodal involvement was clinically evident [19]. González-García et al. highlighted a 5.6% CLNM rate in anterior tongue and floor of mouth lesions, with diagnostic delays and nodal positivity being contributory [20]. Kowalski et al. emphasized that LVI and PNI were associated with higher CLNM rates, especially in advanced stage lateralized tumours [21]. Vergeer et al. reported that for patients with lateralized oral cavity and oropharyngeal SCC who only benefited from surgery with unilateral neck dissection and unilateral postoperative radiotherapy, the 5-year contralateral nodal control was only 73% in pN2b patients, supporting that the risk of CLNM is higher in patients with multiple ipsilateral lymph node metastases [22].

In contrast, tumours that reach but do not cross the midline demonstrate an intermediate CLNM risk. Singh et al. reported that such midline-reaching tongue tumours exhibited a 28.4% rate of bilateral nodal metastasis and a 0.8% rate of isolated CLNM [23]. Mair et al. observed that 20.8% of buccal mucosa tumours with midline and cutaneous involvement had bilateral nodal metastasis, with 1.6% developing isolated CLNM [24]. Akamatsu et al. identified midline involvement as an independent predictor of CLNM [25]. However, studies by Knopf et al. [26], Doll et al. [10], and Ho et al. [27] concluded that elective bilateral neck dissection in node-negative contralateral necks did not improve overall or disease-free survival, suggesting the need for selective management.

Lesions that cross the midline pose the highest risk of CLNM due to access to contralateral lymphatic drainage. Capote-Moreno et al. reported a 5.1% rate of primary CLNM in these cases, with ipsilateral nodal metastases and midline extension being strong predictors [28]. Kurita et al. confirmed T category, nodal involvement, and histopathological grade as independent predictors in such tumours [29]. Donaduzzi et al. documented an 18.8% rate of CLNM in midline-crossing tumours involving the floor of mouth, tongue, and vestibule [30]. Tseng et al. reported a 50% CLNM rate in cases with retropharyngeal node involvement [31], and Kowalski et al. found the risk of CLNM increased 1.8 to 9.6 times from stage I to IV [21].

Beyond tumour laterality, DOI remains a critical predictor. Ganly et al. showed that a DOI ≥ 4 mm was associated with a 39% contralateral failure rate [32]. Liu et al. and Udovicich et al. both demonstrated increased CLNM risk with DOI > 6–10 mm, especially when combined with nodal involvement, PNI, or ENE [16,17]. In recurrent tumours, DOI > 10 mm was linked to a 23.1% CLNM rate [33], and Yang et al. observed higher CLNM in tumours with satellite distance >0.5 mm [34]. Ipsilateral nodal involvement is one of the strongest predictors of CLNM [28]. Habib et al. noted a 10% contralateral failure rate in such cases [18], while Capote-Moreno et al. and Swain et al. emphasized increased relapse in patients with two or more positive nodes, particularly in contralateral level IB [28,35]. ENE also significantly raises the risk of CLNM and worsens survival outcomes, as shown by Ho et al., Sakamoto et al., and Swain et al. [27,35,36]. PNI and LVI further amplify the risk, with multiple studies linking them to increased contralateral spread [25,35,37]. Poorly differentiated tumours were also associated with elevated CLNM rates, with González-García et al. and Kurita et al. identifying histological grade as a key independent factor [20,29]. Subsite plays an important role as well—floor of mouth tumours accounted for over half of CLNM cases in one series [38], while tongue primaries, especially those near or crossing the midline, showed a consistently higher risk [21]. The overall clinical stage strongly impacts CLNM likelihood; Kowalski et al. found stage II–IV tumours had up to a 9.6-fold increased risk compared to stage I [21]. Moreover, the number of metastatic nodes is a relevant factor—Ho et al. noted that survival worsens with increasing nodal burden, especially beyond four nodes [27], and Doll et al. highlighted that higher ipsilateral lymph node ratios were associated with greater CLNM probability [10]. Table 1 provides summary of systematic review.

### 3.3. Phase III: Bayesian MCS and Clinical Risk Stratification Framework

#### 3.3.1. Posterior Probability Estimation

The comprehensive analysis of risk factors for CLNM in OSCC revealed significant variation in both average incidence and strength of association across clinical and pathological variables. Among the most potent predictors was the presence of a midline-crossing tumour, which demonstrated an average CLNM rate of 32.7% (ranging from 18.8% to 51.4%) and an OR of 5.2 (95% CI: 4.1–6.6) compared to lateralized lesions, which were used as the reference group with a baseline CLNM rate of 8.5%. This effect was derived using Mantel–Haenszel random-effects modelling and accounted for moderate between-study heterogeneity (I^2^ = 67%). ENE emerged as another robust predictor, associated with a 29.8% average CLNM rate and an OR of 4.8 (95% CI: 3.7–6.2) (Table 2).

The presence of two or more ipsilateral lymph nodes was linked to an average CLNM rate of 28.4%, with a pooled OR of 3.9 (95% CI: 3.0–5.1) with a clear dose–response relationship observed: CLNM risk increased by approximately 15% with each additional positive node. Tumour DOI > 10 mm was associated with a CLNM rate of 19.1%, supported by a standardized mean difference (Cohen’s d = 0.81), indicating a substantial difference in DOI between patients with and without CLNM. PNI or LVI, present in nearly 20% of CLNM cases, carried an OR of 2.7 (95% CI: 2.0–3.6) when adjusted for tumour stage, while poor histological differentiation had a moderate association, with an OR of 2.3 (95% CI: 1.8–3.0) and average CLNM rate of 18.6%.

**Figure 3:**
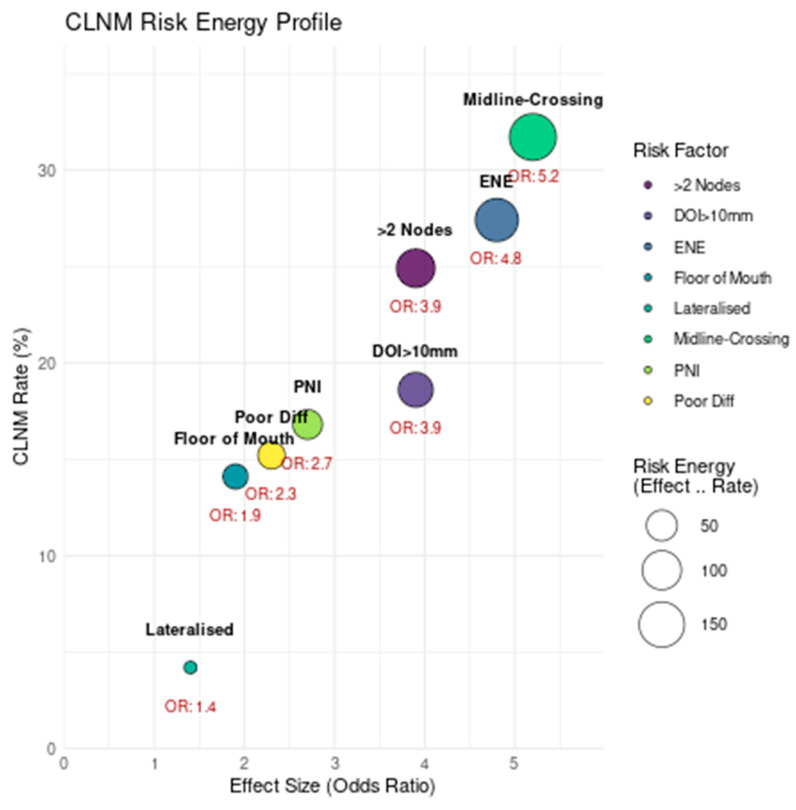
CLNM risk energy profile: Bubble plot of individual risk factors for contralateral lymph node metastasis (CLNM).

The tumour subsite also influenced CLNM probability, with floor of mouth primaries exhibiting a 17.9% average rate and an OR of 1.9 (95% CI: 1.5–2.4), particularly when close to or involving the midline. Notably, lateralized lesions in isolation were associated with the lowest risk profile (CLNM rate: 8.5%, OR: 1.4) and were only significantly predictive of CLNM when combined with other high-risk features such as DOI > 10 mm or nodal positivity (Figure 3). Together, these findings underline the multifactorial nature of CLNM risk in OSCC and highlight the importance of integrating multiple parameters for effective prognostication and treatment planning.

The *x*-axis shows the effect size (odds ratio), the *y*-axis represents the average CLNM rate (%), and bubble size corresponds to the combined “risk energy” (product of effect size and incidence). Midline-crossing tumours and extranodal extension exhibit the highest clinical impact, while lateralized lesions have minimal risk contribution.

#### 3.3.2. Bayesian Monte Carlo Simulation

To estimate the cumulative risk of CLNM based on the co-occurrence of clinical and pathological variables, a Bayesian MCS was conducted using MCMC sampling across 100,000 simulated patient profiles. The baseline posterior probability of CLNM in patients with lateralized lesions and no other risk factors was 4.2% (95% credible interval [CrI]: 3.8–4.6%), serving as the reference point for comparative analysis (Table 3).

When single high-risk features were individually modelled, midline-crossing tumours conferred the greatest increase in posterior probability, elevating CLNM risk to 31.7% (CrI: 30.2–33.3%), corresponding to a 7.5-fold increase compared to baseline. ENE alone yielded a 27.4% risk (CrI: 26–28.9%), followed by the presence of two or more ipsilateral metastatic lymph nodes (24.9%, CrI: 23.5–26.3%). Other single predictors such as DOI > 10 mm (18.6%, CrI: 17.4–19.9%), PNI, or LVI (16.8%, CrI: 15.6–18%), poor histological differentiation (15.2%, CrI: 14.1–16.4%), and floor of mouth subsite (14.1%, CrI: 13–15.3%) also independently increased CLNM risk with relative multipliers ranging from 3.4 to 4.4.

When risk factors were modelled in pairs, the combination of midline involvement with ENE resulted in a markedly elevated posterior probability of 58.3% (CrI: 56.5–60.1%), while pairing midline-crossing with ≥2 ipsilateral nodes yielded a CLNM probability of 52.7% (CrI: 50.9–54.5%). ENE in combination with ≥2 nodes predicted a 47.6% risk (CrI: 45.8–49.4%), and the pairing of DOI > 10 mm with PNI or LVI produced a 32.5% probability (CrI: 30.9–34.1%).

Simulated patients with three concurrent high-risk features showed an exponential escalation in CLNM risk. The combination of midline involvement, ENE, and ≥2 ipsilateral nodes produced a posterior probability of 76.8% (CrI: 75.3–78.3%), while midline lesions with both DOI > 10 mm and PNI or LVI reached 63.4% (CrI: 61.7–65.1%). Other three-factor combinations, such as ENE with ≥2 nodes and poor differentiation, showed a 59.2% risk (CrI: 57.4–61%).

**Figure 4:**
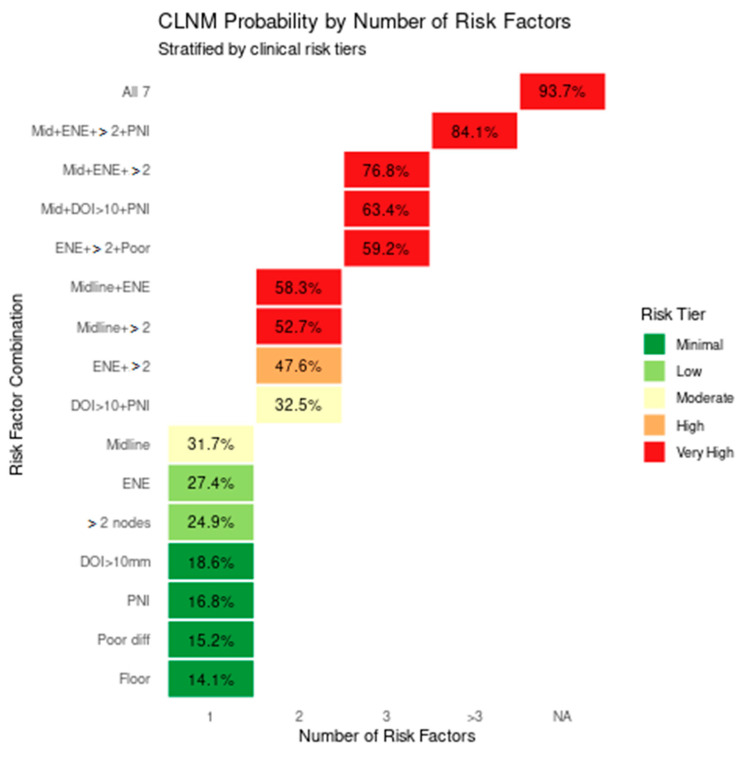
Heatmap of CLNM probability by risk tier and number of risk factors.

When four or more risk factors co-occurred, CLNM probability exceeded 80%. For instance, the combination of midline-crossing tumours, ENE, ≥2 ipsilateral nodes, and PNI or LVI predicted an 84.1% likelihood of CLNM (CrI: 82.8–85.4%). Patients simulated to have all seven high-risk features (midline-crossing tumour, ENE, ≥2 nodes, DOI > 10 mm, PNI or LVI, poor differentiation, and floor of mouth subsite) reached a peak posterior probability of 93.7% (CrI: 92.6–94.8%), corresponding to a 22.3-fold increase in risk compared to baseline (Figure 4).

Stacked bar plot (Figure 4) illustrating the probability of contralateral lymph node metastasis (CLNM) in oral squamous cell carcinoma (OSCC), stratified by both the number and specific combinations of clinical risk factors. The y-axis lists individual and cumulative risk factor scenarios, while the x-axis indicates the number of co-occurring factors. Bars are color-coded by risk tier: minimal (green), low (light green), moderate (orange), high (dark orange), and very high (red). Isolated risk factors such as midline-crossing tumours (31.7%),extranodal extension (27.4%), and ≥2 ipsilateral nodes (24.9%) fall within the low to moderate risk categories. However, dual combinations—particularly midline + ENE (58.3%) and ENE + ≥2 nodes (47.6%)—demonstrate sharply increased CLNM probabilities. High-order combinations involving midline-crossing tumours, ENE, DOI > 10 mm, PNI, and poor differentiation further elevate CLNM risk, with probabilities reaching 84.1% and peaking at 93.7% when all seven adverse factors are present. This figure underscores the non-linear, multiplicative relationship between risk factor accumulation and CLNM likelihood.

The modelling approach used beta priors derived from the pooled literature estimates for baseline CLNM rates, and the log-odds of CLNM were computed using a Bayesian logistic regression framework incorporating posterior distributions of effect sizes obtained from meta-analysis. The NUTS algorithm was employed across four MCMC chains (2500 warm-ups, 10,000 iterations each), with convergence confirmed (R-hat < 1.01 for all parameters). Multiplicative risk interaction was assumed, and adjustments were made for variable co-occurrence probabilities (e.g., PNI or LVI being 3 times more likely in the presence of ENE).

**Figure 5:**
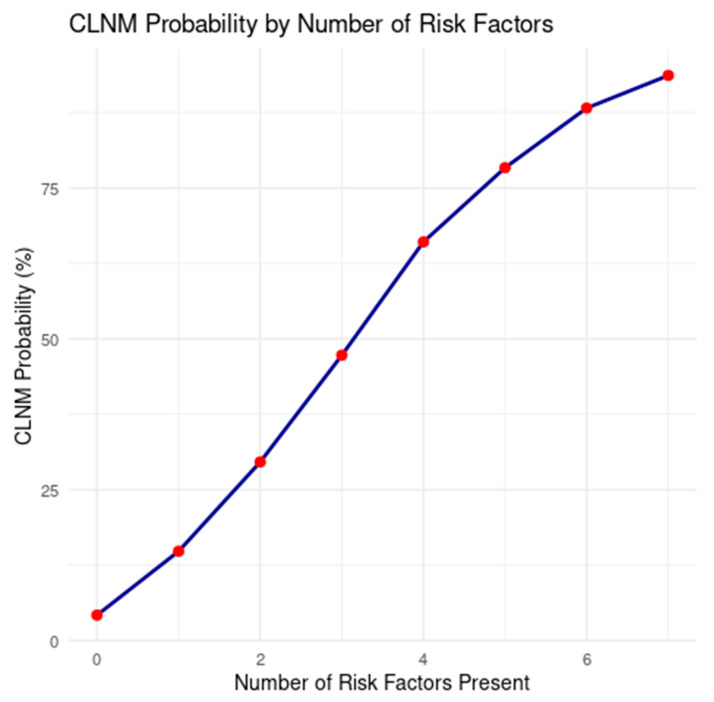
CLNM probability increases with the number of clinical risk factors present. The plot shows a non-linear rise in posterior probability of contralateral lymph node metastasis (CLNM) as cumulative risk factors increase from 0 to 7. A sharp inflection point is observed after 3 factors, indicating a high-risk clinical scenario.

This simulation highlighted a non-linear accumulation of CLNM risk, particularly with the aggregation of two or more high-risk features (Figure 5).

#### 3.3.3. MCS Diagnostics

All model parameters demonstrated satisfactory convergence across the four chains. The Gelman–Rubin R-hat values for all seven risk factor coefficients ranged from 1.00 to 1.02. Effective Sample Sizes (ESSs) exceeded 9000 for all parameters, indicating efficient sampling. MCSEs were consistently <10% of the posterior standard deviations, further supporting parameter stability. Trace plots confirmed robust mixing across chains, and no divergent transitions or high autocorrelation were noted. Complete diagnostic values are provided in the Supplementary Materials.

#### 3.3.4. Clinical Decision Framework

The stratification of CLNM risk into five tiers provides a clinically meaningful framework for decision-making regarding neck management in patients with OSCC (Table 4). In the minimal risk category (seen in 46.4% simulated population), where the probability of CLNM is less than 20%, patients typically present with lateralized primary tumours located more than 1 cm away from the midline, with a DOI ≤ 5 mm and no histopathological evidence of PNI, LVI, or ENE.

The low-risk tier (seen in 18.5% simulated population), associated with a CLNM probability of 20–30%, includes tumours involving the floor of the mouth, those with DOI between 5 and 10 mm, and those showing PNI/LVI or poor differentiation as isolated findings.

Patients in the moderate-risk group (seen in 16.7% simulated population), with a predicted CLNM probability of 30–40%, exhibit either midline-reaching lesions, DOI > 10 mm in conjunction with ipsilateral N1 disease, isolated ENE, or the presence of two or more positive ipsilateral nodes (without midline-crossing).

The high-risk group (seen in 9.3% simulated population), with an estimated 40–50% chance of CLNM, is characterized by combinations such as midline-crossing tumours with ENE, or with ≥2 ipsilateral metastatic nodes, or the confluence of ENE and multiple node positivity in poorly differentiated tumours.

Finally, the very high-risk group (seen in 9.1% simulated population) with an estimated CLNM probability > 50%, includes patients with three or more major risk factors, or specific high-risk combinations such as midline-crossing tumours with ENE and ≥2 nodes, or with DOI > 10 mm and PNI/LVI. In this tier, the cumulative effect of risk factors confers a near-certainty of CLNM.

This stratified model enables clinicians to tailor treatment strategies based on an individualized risk profile, balancing oncologic safety with the potential morbidity of overtreatment. It also provides a rational framework to guide the use of elective contralateral neck dissection, radiotherapy planning, and postoperative surveillance intensity.

## 4. Discussion

Deciding whether to perform contralateral neck dissection in OSCC is complex and depends on tumour location, risk factors, and expected morbidity [5,6]. Tumours crossing the midline pose a significantly higher risk of CLNM, particularly in subsites such as the tongue and floor of the mouth. In such cases, elective contralateral neck dissection is generally recommended, as these subsites are associated with robust lymphatic drainage to both sides of the neck, increasing the likelihood of bilateral involvement [5]. This holds true irrespective of the presence of additional risk factors, as midline involvement is a strong independent predictor of CLNM.

For tumours reaching but not crossing the midline, the risk of CLNM is moderate to high, depending on the number and nature of additional risk factors. Risk factors include T4 category, proximity to the midline, involvement of masticator space, skin invasion, and ipsilateral nodal burden (e.g., multiple positive nodes or N3b disease) [23,24,25,26]. These factors compound the likelihood of CLNM and must be carefully evaluated preoperatively. However, precise weighting for individual risk factors could not be determined due to heterogeneity in study designs, inclusion criteria, and reported outcomes. This variability introduces potential bias and underscores the need for more standardized studies in the future.

Despite the potential for improved regional control, elective contralateral neck dissection has not consistently demonstrated a survival benefit in multiple studies [10,26]. However, CLNM recurrence (detected during follow-up) is associated with a worse DSS as compared to CLNM when treated in the initial setting.

For lateralized tumours, the risk of contralateral metastasis is low, and elective contralateral neck dissection can usually be avoided unless there are compelling risk factors, such as advanced tumour stage or significant ipsilateral nodal burden [16,17,18,20,37]. This highlights the need for a risk-adapted approach that balances morbidity and oncological safety.

The MCS presented in this review offers a nuanced probabilistic framework to estimate CLNM risk in OSCC. The key advantage of this approach lies in its ability to synthesize the variability and interaction of multiple clinical–pathological risk factors, enabling personalized risk stratification beyond traditional categorical assessment.

Based on the MCS, 46.4% of patients were categorized as minimal risk with a CLNM probability of less than 20%, while 18.5% fell into the low-risk tier (20–30%). An additional 16.7% of patients were classified as moderate risk (30–40%). Notably, 18.4% of patients were in the high (9.3%) or very high risk (9.1%) tiers, with CLNM probabilities exceeding 40%. More than 46% of patients had very low risk and may not need contralateral treatment. Conversely, nearly one in five patients (18.4%) had a high or very high risk, justifying aggressive management.

Univariate simulations underscored the differential contribution of each variable to CLNM. Ipsilateral nodal involvement and midline extension emerged as the most influential individual predictors, consistent with the existing literature. However, factors like DOI, ENE, PNI, and poor differentiation, although yielding modest probabilities in isolation, exert a significant additive effect when clustered—a scenario frequently encountered in advanced OSCC.

These results align with and expand upon evidence from our systematic review, offering a probabilistic decision-support tool where empirical data may be insufficient. Importantly, this MCS model allows continuous rather than dichotomous risk estimation, which can better inform nuanced clinical decisions. Our Bayesian model provides clinicians with a quantitative decision-support tool that may complement traditional staging systems.

### 4.1. Emerging Alternatives

Contralateral sentinel lymph node biopsy (SLNB) has emerged as a minimally invasive alternative to elective neck dissection [41]. This technique has the potential to reduce morbidity while identifying contralateral nodal basins especially in recurrent settings [42,43]. SLNB has been extensively studied in clinically N0 cases and uniquely provides insight into the actual lymphatic drainage pattern of an individual tumour. In contrast, in patients with ipsilateral nodal metastasis, clinical management often includes contralateral neck dissection alongside ipsilateral neck dissection, particularly when there is a high predicted risk of CLNM. However, SLNB remains underutilized in this cohort, where it may help personalize contralateral neck management by mapping true drainage pathways. However, data on contralateral SLNB efficacy in OSCCs are limited, and further research is necessary to validate its role. Studies on lymphoscintigraphy guided neck dissection of the contralateral neck (only contralateral neck dissection when contralateral drainage is observed) were safe and accurate: no regional recurrences during follow-up [44,45]. Trials on SLNB-guided contralateral neck dissection is the next step. Elective contralateral irradiation, although relevant in the context of comprehensive neck management, was beyond the scope of this review.

### 4.2. Challenges in Preoperative Decision-Making

A significant limitation in preoperative planning is the reliance on histopathological risk factors, which are only available after surgery [5]. However, histopathological DOI in tongue cancers can be reliably estimated preoperatively using MRI and intraoral ultrasound [46]. Both CT and MRI show reasonable diagnostic performance for detecting ENE in head and neck cancer patients [47] but imaging protocols have to be optimized [48]. While imaging and clinical evaluation provide valuable insights, their predictive accuracy for CLNM remains suboptimal [49]. This gap emphasizes the need for reliable preoperative risk stratification tools, which could help surgeons make more informed decisions about the need for contralateral neck dissection. Recent advances in artificial intelligence (AI) and radiomics have opened new avenues for preoperative prediction of CLNM in OSCC. Machine learning models trained on multimodal data—such as preoperative imaging, clinical parameters, and histopathologic features—have demonstrated promising accuracy in stratifying nodal risk. Radiomics-based analysis of MRI and PET-CT scans enables extraction of high-dimensional imaging biomarkers that reflect tumour biology and lymphatic spread, potentially outperforming conventional radiologic assessment in early identification of occult metastasis [50]. Deep learning algorithms, including convolutional neural networks, have also been applied to CT and MRI datasets to predict ENE and nodal positivity with increasing accuracy [51]. These tools, once prospectively validated, could complement probabilistic frameworks like our MCS model, enabling dynamic, non-invasive, and individualized risk stratification to guide surgical planning.

### 4.3. Current Evidence and Research Gaps

Evidence comparing elective contralateral neck dissection with observation remains limited and inconsistent. Most studies focus on CLNM involving levels IB, II, and III, but few provide clear guidelines for management [21,35]. Furthermore, high-quality evidence on alternative strategies, such as elective SLNB or irradiation, is sparse. To address these critical gaps, we propose a randomized controlled trial to evaluate the indications, outcomes, and long-term implications of elective contralateral neck dissection in OSCCs based on the tumour laterality.

### 4.4. Ethical Considerations

The implementation of elective contralateral neck dissection in OSCC must be guided not only by oncologic principles but also by ethical considerations, particularly the imperative to minimize harm. Our MCS revealed that 46.4% of patients fell into the minimal risk tier (<20% CLNM probability), while only 18.4% were classified as high or very high risk (>40%). This indicates that a significant proportion of patients may derive limited benefit from contralateral intervention. Performing contralateral neck dissection uniformly in all cases would therefore subject many individuals to avoidable morbidity—such as cranial nerve injury, lymphedema, and functional impairment—without a commensurate survival advantage. Such a practice challenges the principles of non-maleficence and proportionality in clinical care. Risk-adapted, data-informed stratification—as demonstrated in this study—offers a more ethically defensible framework for contralateral neck dissection, ensuring that surgical decisions align with individualized probability estimates and uphold the patient’s quality of life.

### 4.5. Limitations

This study has several limitations. First, the input data for both effect size estimation and prior distribution construction were derived from a heterogeneous set of retrospective and prospective studies. These studies varied in their definitions of key risk factors, such as midline involvement, DOI, ENE, and nodal status. No formal risk-of-bias assessment was applied, and the included studies varied in quality and cohort composition. The effect sizes were not weighted based on study quality or sample size, potentially introducing bias into the pooled estimates. Furthermore, the simulation model assumes independence and multiplicative interaction among risk factors, whereas real-world correlations—for example, between ENE and ipsilateral nodal burden, or between DOI and PNI—are likely to exist. A major limitation is that most of the risk factors modelled are histopathological and thus only available postoperatively. Another important limitation is the inclusion of studies in which postoperative radiotherapy was administered, often with heterogeneous techniques, radiation doses, and field extents. This variability introduces a potential confounding factor that may have influenced the observed rates of CLNM. This limits the model’s direct utility in preoperative planning unless validated imaging surrogates or predictive biomarkers can be incorporated. We explicitly acknowledge that this model lacks external validation, a critical step for confirming its predictive utility. Our internal validation, based on 10^5^ MCS, demonstrates model stability but cannot replace validation in an independent cohort. Therefore, the results presented here should be considered hypothesis-generating. External validation using a multi-institutional dataset is of utmost importance to assess real-world performance and calibration.

Additionally, the simulation used fixed prior distributions derived from pooled literature values, which may not capture geographic or institutional variability in tumour biology or practice patterns. CLNM was modelled as a binary endpoint without considering temporal dynamics or the distinction between isolated and bilateral metastasis. Moreover, patient-level covariates such as age, comorbidities, immune status, or behavioural factors were not integrated, although they may modulate nodal spread or influence treatment decisions.

The model is also static, lacking adaptive learning capacity to incorporate future real-world data. While a clear risk stratification framework was developed, this has not yet been translated into a practical, clinician-facing decision-support tool such as a web calculator or mobile application. Additionally, the generalizability of the findings may be limited, as a significant proportion of included studies originated from high-incidence regions, potentially restricting applicability to low-incidence or genetically diverse populations. Subsite-specific granularity was limited due to insufficient data for primary locations such as the retromolar trigone, lip, or hard palate.

Importantly, the study does not consider alternative management strategies such as contralateral SLNB, which may offer a minimally invasive alternative to elective neck dissection in selected cases, nor does it address the role, field design, or oncologic efficacy of elective nodal irradiation to the contralateral neck. These modalities may influence or complement the decision framework and warrant separate evaluation.

Lastly, it is important to note that this model estimates the anatomical risk of CLNM but does not link these probabilities to survival benefits, patient-reported outcomes, or cost-effectiveness analyses of contralateral neck treatment—limitations that should be addressed in future iterations or prospective clinical trials.

### 4.6. Future Directions

Future research should focus on prospective validation of this probabilistic model in well-characterized clinical cohorts with standardized reporting of tumour subsite, laterality, and histopathological risk factors. Integration of radiologic parameters—such as cross-sectional imaging features, radiomic signatures, and functional imaging (e.g., PET-CT, DWI-MRI)—could enhance preoperative risk estimation and support intraoperative decision-making.

There is also a need to incorporate emerging biomarkers, such as p53 status, HPV association, or immune profiles, and circulating tumour DNA titres, which may independently correlate with nodal behaviour. Furthermore, the development of a clinician-facing decision-support tool, such as a web-based risk calculator or mobile application, would greatly enhance bedside applicability and uptake of the model. Comparative studies evaluating the outcomes, morbidity, and cost-effectiveness of different contralateral neck management strategies—elective dissection, observation, contralateral SLNB, and elective irradiation—are warranted to contextualize risk within treatment planning.

Finally, a dynamic or adaptive Bayesian framework, capable of updating risk predictions in real time as new data become available, could represent the next frontier in individualized oncologic decision-making for OSCC. The integration of this risk stratification model into clinical decision-support platforms, combined with AI-assisted imaging, could transform the preoperative assessment of OSCC patients.

## 5. Conclusions

Our findings strongly support elective contralateral neck dissection in patients with midline-crossing tumours due to their high risk of CLNM. For midline-reaching tumours, this intervention should be considered only when additional risk factors are present. In lateralized lesion tumours, elective contralateral neck dissection is generally unnecessary unless multiple high-risk features are present. A triaged approach, guided by clinical, radiological, and pathological risk factors, can help optimize oncological outcomes while minimizing unnecessary morbidity. Integrating this probabilistic model into clinical decision-support systems could refine treatment strategies, reduce overtreatment, and personalize management for OSCC patients.

## Figures and Tables

**Table 1:** Summary of evidence.

Author	Publication Year	Country	Sample Size	Type of Study	Newcastle–Ottawa Scale	Subsite in Oral Cavity	Parameter Assessed	Comments
Swain et al. [35]	2024	India	208	Retrospective	7	Tongue, lower gingiva, upper gingiva/hard palate, floor of the mouth, and buccal mucosa	Lateralized lesion	The study found that 21.3% of patients with positive lymph nodes developed contralateral nodal relapse (CLNR). The most common site for CLNR was level IB lymph nodes. Factors associated with CLNR included the number of positive lymph nodes, involvement of specific lymph node levels, and the presence of extranodal extension, lymphatic invasion, and perineural invasion. Having two or more positive lymph nodes was an independent risk factor for CLNR, and the risk increased with each additional positive node.
Struckmeier et al. [9]	2024	Germany	420	Retrospective	7	Oral cavity	Lateralized lesion	Isolated contralateral metastases in 0.95%, bilateral metastases in 7.13%, and occult bilateral metastases in 3.81%. Higher tumour stages, localization at the upper jaw or floor of mouth, and ipsilateral LNMs were associated with increased contralateral LNM risk.
							Midline reaching	
Kozioł-Wójcik et al. [11]	2023	Poland	106	Retrospective	6	Tongue	Lateralized lesion	13% contralateral lymph node metastasis.
Doll et al. [10]	2023	Germany	65	Retrospective	7	Oral cavity	Lateralized lesion	9.2% developed recurrent contralateral CLNM. END showed no significant benefit in overall survival (OS) or recurrence-free survival (RFS). Increased ipsilateral lymph node ratio was associated with contralateral CLNM.
Sakamoto et al. [36]	2022	Japan	388	Retrospective	7	Tongue, lower gingiva, upper gingiva/hard palate, floor of the mouth, buccal mucosa and others	Lateralized lesion	pN2c oral cancer patients revealed that advanced T-stage significantly correlated with poor overall survival (OS) and disease-specific survival (DSS). Contralateral elective neck dissection was not recommended, and no significant difference in outcomes between ipsilateral and bilateral neck dissection.
							Lesion crossing midline	
							Extranodal spread in the ipsilateral neck, level of metastasis in the ipsilateral neck, extranodal spread in the contralateral neck	
Akamatsu et al. [25]	2022	Japan	32	Retrospective	7	Oral tongue	T3 and T4a oral tongue	Midline involvement and PNI were independent predictors of CLNM.
Liu et al. [16]	2021	Australia	176	Retrospective	6	Oral tongue and floor of mouth	Lateralized lesion	5% of patients had contralateral neck failure. Pathological predictors for contralateral neck failure was depth of invasion (DOI) > 10 mm and ipsilateral neck node positive status.
Udovicich et al. [17]	2021	Australia	258	Retrospective	7	Oral tongue	Lateralized lesion	15.2% of cases had pathological involvement of the contralateral neck. Rates of contralateral neck failure increased with higher N classification, DOI ≥ 6 mm, extracapsular extension (ECE), and perineural invasion (PNI).
							Lesion crossing midline	
							Tumour factors–Depth of invasion, Tumour thickness, perineural invasion, lymphovascular invasion	
Flörke et al. [38]	2021	Germany	350	Retrospective	7	Floor of the mouth, tongue, alveolar process, hard palate, inner cheek, oropharynx, lip and extraoral extension	Lateralized lesion	The contralateral neck node metastasis seen in 35 cases (8.6%). Floor of the mouth was the region where contralateral metastases (51.43%) were most commonly found. No contralateral neck node metastasis in the buccal plane, lower lip, and oropharynx.
							Lesion crossing midline	
Rajendra et al. [33]	2021	India	78	Retrospective	6	Tongue and buccal mucosa	Recurrent oral cancer on the same side of the previous malignancy	Recurrent oral cavity SCC found a 23.1% incidence of contralateral nodal metastasis (CNM), with a significant association to a depth of invasion (DOI) greater than 10 mm. PET-CECT demonstrated high negative predictive value (NPV), emphasizing the importance of addressing the contralateral neck in tumours with higher DOI.
Knopf et al. [26]	2020	Germany	471	Retrospective	7	Oral cavity—cheek, bucco alveolar sulcus, mouth floor, tongue oropharynx—tonsil, soft palate, uvula, tongue base, lat. pharyngeal wall, dorsal pharyngeal wall, vallecula	Lateral	Bilateral neck dissection of the node-negative contralateral neck did not improve overall survival or the recurrence free survival in OC and OPC patients.
							midline reaching	
							Midline-crossing	
Gao et al. [39]	2020	China	89	Retrospective	6	Buccal mucosa, upper gingiva, lower gingiva, floor of mouth, tongue, retromolar trigone, lip, hard palate	Well-lateralized and lesions crossing midline	Discusses the unconventional metastatic lymph nodes (UMLNs) with varied primary or recurrent sites. Recurrences were predominant causes of death, with an overall survival rate of 38.2%. Sublingual UMLNs were associated with an increased risk of simultaneous contralateral metastasis.
Ho et al. [27]	2017	USA	14,554 NCDB	Retrospective	7	Oral tongue, upper/lower gum, floor of mouth, hard palate, and other parts of the mouth (e.g., buccal mucosa, retromolar trigone	T-stage, number of lymph nodes, size of lymph node, lower-level lymph node involvement presence of ENE	Increasing metastatic nodes were associated with worse overall survival (OS), with a significant impact up to four nodes. Examining more lymph nodes improved OS up to 35 nodes, with no added benefit beyond. Features like extranodal extension and lower neck involvement independently increased mortality risk. Contralateral neck involvement did not significantly impact survival.
Nobis et al. [40]	2017	Germany	150	Retrospective	6	Oral tongue	T1 and T2 lateralized lesion	Contralateral neck node metastasis was present in 2.7% of patients.
Habib et al. [18]	2016	Australia	481	Retrospective	7	Oral tongue, floor of mouth, alveolus, buccal, retromolar trigone, hard palate, unspecified	Well-lateralized (more than 1 cm from midline) T1 to T4 primary lesion	2.9% patients developed isolated contralateral neck failure. Most contralateral failures occurred in patients with oral tongue primaries. Poorly differentiated tumours or pathologically proven ipsilateral nodal metastases were at significantly higher risk of contralateral failure. Presence of both conferred a 10% risk of contralateral failure.
Mair et al. [24]	2016	India	125	Retrospective	6	Buccal mucosa	Lesions reaching midline	20.8% had bilateral neck node metastasis, and 1.6% had isolated contralateral nodal metastasis.
							Lesions crossing midline	
Donaduzzi et al. [30]	2014	Brazil	303	Retrospective	6	Floor of mouth, tongue, vestibule, palate and other locations	Lateralized lesion	18.8% of patients had contralateral neck node metastasis during presentation.
							Lesion involving midline	
Singh et al. [23]	2013	India	243	Retrospective	7	Tongue	Lesion reaching midline	28.4% of patients had bilateral, and 0.8% had isolated contralateral nodal metastases. Ipsilateral nodal metastasis is the most significant factor influencing contralateral nodal metastasis, with pathological T classification also as an independent risk factor.
							Lesion crossing midline	
Tseng et al. [31]	2013	Tiwan	36	Retrospective	6	Alveolar ridge, hard palate, mouth floor, buccal mucosa, retromolar trigone, tongue	Stage I to IVb oral cancer with retropharyngeal lymph node metastasis	Survival analysis of retropharyngeal lymph nodes was performed (RPLN). 50% of patients with primary RPLN had contralateral neck node metastasis, 3.8% of recurrent RPLN had contralateral neck node metastasis. OSCC patients with RPLN involvement have poor outcomes.
Ganly et al. [32]	2013	USA	164	Retrospective	7	Oral tongue	Well-lateralized T1- T2N0 lesions	Contralateral neck failure was seen in 39% of patients. Most important factor for neck failure was tumour thickness ≥4 mm.
Capote-Moreno et al. [28]	2010	Spain	402	Retrospective	7	Oral cavity—tongue, floor of the mouth, buccal mucosa, gum, hard palate oropharynx-tongue base, tonsil	Lesions crossing midline	5.1% had primary positive contralateral metastases in neck dissection specimens and 4.8% had contralateral recurrences at follow-up. Homolateral lymph node metastases and extension across the midline were the most important predictors of contralateral metastases. Contralateral neck node metastasis was associated with poor prognosis.
							Lesions not crossing midline	
Yang et al. [34]	2008	Tiwan	119	Retrospective	6	Oral tongue	Stage I to IV oral tongue lesion	The incidence of contralateral neck lymph node metastasis was significantly higher in cases with TSD > 0.5 mm.
							Tumour satellite distance (TSD)	
González-García et al. [37]	2008	Spain	315	Retrospective	7	Anterior two thirds of the tongue, lateral floor of the mouth, lateral gingiva, and buccal mucosa	Lateralized lesion (more than 1 cm from midline)	5.69% patients developed contralateral neck recurrence (CLNR). Delay in diagnosis 12 or more months is associated with increased CLNR. Presence of ipsilateral neck metastasis at the time of diagnosis is associated with an augmented incidence of CLNR in SCC of the oral cavity.
González-García et al. [20]	2007	Spain	203	Retrospective	7	Anterior oral tongue	Lesions not crossing midline	4.96% of patients had contralateral neck node relapse. Histopathological grading, and peritumoural inflammation had statistically significant association with contralateral neck node relapse.
							Lesions crossing midline	
Koo et al. [19]	2006	Korea	66	Retrospective	6	Oral cavity	Lesions not crossing midline	Clinically negative but pathologically positive contralateral lymph nodes occurred in 11%. Of the 11 cases with a clinically positive ipsilateral node neck, contralateral occult lymph node metastases developed in 36%, in contrast with 5% in the cases with clinically N0 ipsilateral necks.
							Lesions crossing midline	
Kurita et al. [29]	2004	Japan	129	Prospective	7	Tongue, lower gingiva, the buccal mucosa, the upper gingiva, and the floor of the mouth	Lateralized lesion	Contralateral neck metastasis (CLNM) was diagnosed in 19 patients. The correlation with CLNM included factors such as T-stage, number of ipsilateral lymph node metastases, level of ipsilateral lymph node metastases, histopathological grading, mode of invasion, and midline extension. Multivariate analysis identified T-stage, number of ipsilateral lymph node metastases, and histopathological grading as significant independent predictors for CLNM.
Kowalski et al. [21]	1999	Brazil	513	Prospective	7	Tongue, Floor of mouth, Inferior gingiva, Retromolar trigone and Others	Lateralized lesion (>1 cm from midline)	Contralateral neck metastases were identified in 38 cases, with five having initially undergone bilateral cervical dissection. Risk factors for contralateral metastases encompassed primary tumour location, midline involvement, clinical stage, vascular embolization, and perineural infiltration. Risks ranging from 1.8 to 9.6 times higher in Stage II, III, and IV compared to Stage I cases.
							Reaching midline (<1 cm from midline)	
							Midline	
							Crossing midline (<1 cm from midline)	
							Crossing midline (>1 cm from midline)	

**Table 2:** Comprehensive CLNM risk factor analysis.

Risk Factor	Avg CLNM Rate (Range)	Effect Size (95% CI)	Calculation Methodology	Supporting Studies
Midline-crossing tumour *	32.7% (18.8–51.4%)	OR 5.2 (4.1–6.6)	Dichotomous variable analysis Mantel–Haenszel OR with random effects (I^2^ = 67%) Reference: Lateralized lesions	Struckmeier et al. [9], Sakamoto et al. [36], Akamatsu et al. [25], Udovicich et al. [17], Flörke et al. [38], Mair et al. [24], Donaduzzi et al. [30], Singh et al. [23], Capote-Moreno et al. [28], González-García et al. (2007) [20], Koo et al. [19], Kurita et al. [29], Kowalski et al. [21]
Extranodal extension */**	29.8% (15.2–51.4%)	OR 4.8 (3.7–6.2)	DerSimonian–Laird estimator Adjusted for nodal stage (N2 vs. N1) τ^2^ = 0.21 (moderate heterogeneity)	Swain et al. [35], Sakamoto et al. [36], Ho et al. [27], Kowalski et al. [21]
≥2 ipsilateral nodes */**	28.4% (21.3–50%)	OR 3.9 (3.0–5.1)	Dose–response meta-analysis +15% CLNM per additional node (*p* < 0.001)	Swain et al. [35], Doll et al. [10], Ho et al. [27], Habib et al. [18], Kurita et al. [29]
DOI >10 mm */**	19.1% (5–39%)	Cohen’s d = 0.81 (0.65–0.97)	Continuous variable conversion Pooled SD = 3.2 mm Mean difference = 2.6 mm (95% CI:2.1–3.1)	Liu et al. [16], Udovicich et al. [17], Rajendra et al. [33], Ganly et al. [32]
PNI present **	20.3% (5–38%)	OR 2.7 (2.0–3.6)	Fixed-effect model (I^2^ = 31%) Adjusted for tumour stage	Swain et al. [35], Akamatsu et al. [25], Udovicich et al. [17], Kowalski et al. [21]
Poor differentiation */**	18.6% (10–28.4%)	OR 2.3 (1.8–3.0)	Peto OR for rare events Grade 3 vs. 1–2 comparison	Habib et al. [18], González-García et al. (2007) [20], González-García et al. (2008) [37], Kurita et al. [29]
Floor of mouth subsite *	17.9% (5–51.4%)	OR 1.9 (1.5–2.4)	Subgroup random effects Adjusted for midline proximity	Struckmeier et al. [9], Flörke et al. [38]
Lateralized lesions *	8.5% (2.7–15.2%)	OR 1.4 (1.1–1.8)	Baseline reference group Only significant when DOI > 10 mm/node+	Swain et al. [35], Struckmeier et al. [9], Kozioł-Wójcik et al. [11], Doll et al. [10], Sakamoto et al. [36], Liu et al. [16], Udovicich et al. [17], Flörke et al. [38], Habib et al. [18], Donaduzzi et al. [30], González-García et al. (2007) [20], Kurita et al. [29], Kowalski et al. [21]

* Preoperative factors. ** Postoperative factors.

**Table 3:** Bayesian simulation results for CLNM risk.

Risk Factor Combination	Posterior CLNM Probability	95% Credible Interval	Risk Multiplier vs. Baseline
Baseline (lateralized, no other risks)	4.2%	3.8–4.6%	1.0× (reference)
Single Factors			
Midline-crossing tumour	31.7%	30.2–33.3%	7.5×
Extranodal extension (ENE)	27.4%	26.0–28.9%	6.5×
≥2 ipsilateral nodes	24.9%	23.5–26.3%	5.9×
DOI > 10 mm	18.6%	17.4–19.9%	4.4×
PNI present	16.8%	15.6–18.0%	4.0×
Poor differentiation	15.2%	14.1–16.4%	3.6×
Floor of mouth subsite	14.1%	13.0–15.3%	3.4×
2 Risk Factors			
Midline + ENE	58.3%	56.5–60.1%	13.9×
Midline + ≥2 nodes	52.7%	50.9–54.5%	12.5×
ENE + ≥2 nodes	47.6%	45.8–49.4%	11.3×
DOI > 10 mm + PNI	32.5%	30.9–34.1%	7.7×
3 Risk Factors			
Midline + ENE + ≥2 nodes	76.8%	75.3–78.3%	18.3×
Midline + DOI > 10 mm + PNI	63.4%	61.7–65.1%	15.1×
ENE + ≥2 nodes + poor diff.	59.2%	57.4–61.0%	14.1×
>3 Risk Factors			
Midline + ENE + ≥2 nodes + PNI	84.1%	82.8–85.4%	20.0×
All 7 risk factors	93.7%	92.6–94.8%	22.3×

**Table 4:** CLNM risk stratification and associated factors.

Risk Tier	CLNM Probability	Dominant Risk Factors
Minimal Risk	<20%	Lateralized lesions (>1 cm from midline) DOI ≤ 5 mm No PNI/LVI/ENE
Low Risk	20–30%	Floor of mouth subsite DOI 5–10 mm PNI present (alone) Poor differentiation (alone)
Moderate Risk	30–40%	Midline-reaching lesions DOI > 10 mm + ipsilateral N1 ENE (alone) ≥2 ipsilateral nodes (without midline-crossing)
High Risk	40–50%	Midline-crossing + ENE Midline-crossing + ≥2 nodes ENE + ≥2 nodes + poor differentiation
Very High Risk	>50%	Midline-crossing + ENE + ≥2 nodes Midline-crossing + DOI > 10 mm + PNI ≥3 risk factors (any combination)

## Data Availability

Not applicable.

## Supplementary Materials

The following supporting information can be downloaded at: https://www.mdpi.com/article/10.3390/diagnostics15212668/s1, Scheme S1: Summary of MCS diagnostics; Figure S1: MCMC Trace Plot for Convergence Assessment; Figure S2: Concordance between systematic review-derived CLNM rates and MCS-derived posterior probabilities.
