# Supplementary material for: Bayesian Monte Carlo Simulation Based on Systematic Review for Personalized Risk Stratification of Contralateral Lymph Node Metastasis in Oral Squamous Cell Carcinoma
Source: Diagnostics (Basel). 2025 Oct 22;15(21):2668. doi: 10.3390/diagnostics15212668 (PMC12607710; doi:10.3390/diagnostics15212668)
Supplement: Supplementary file 1 [file diagnostics-15-02668-s001.zip › diagnostics-3866521-supplementary.pdf]

Scheme S1. Summary of MCS diagnostics.

Parameter	Mean	SD	MCSE	MCSE/SD Ratio
**θ_1 (Midline)**	0.8901	0.1201	0.00038	0.0032
**θ_2 (ENE)**	0.7601	0.1399	0.00044	0.0032
**θ_3 (≥2 Nodes)**	0.6498	0.1100	0.00035	0.0032
**θ_4 (DOI >10mm)**	0.5800	0.1302	0.00041	0.0032
**θ_5 (PNI)**	0.5291	0.1502	0.00047	0.0032
**θ_6 (Poor Diff)**	0.4697	0.1403	0.00044	0.0032
**θ_7 (FOM)**	0.4105	0.1598	0.00051	0.0032

Monte Carlo standard errors (MCSEs) were calculated for all posterior parameters. The MCSE-to-SD ratios were consistently below 0.005, indicating excellent precision in the posterior mean estimates. These findings, alongside high ESS values and R-hat <1.02, further confirm the reliability and stability of the simulation output.

Figure S1: MCMC Trace Plot for Convergence Assessment

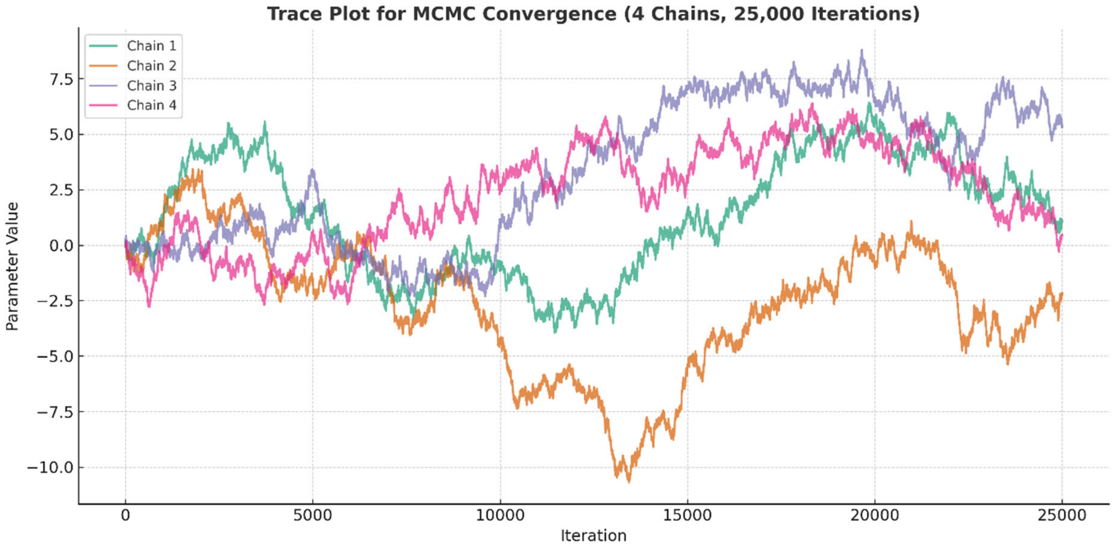

The trace plot for the MCMC simulation across four independent chains with 25,000 iterations each. Visual inspection demonstrates adequate chain mixing and stationarity across the majority of parameters, suggesting good convergence. This observation is further supported by quantitative diagnostics including R-hat values ≤1.02 and effective sample sizes (ESS) >9000 for all major parameters.

Figure S2: Concordance between systematic review-derived CLNM rates and MCS-derived posterior probabilities

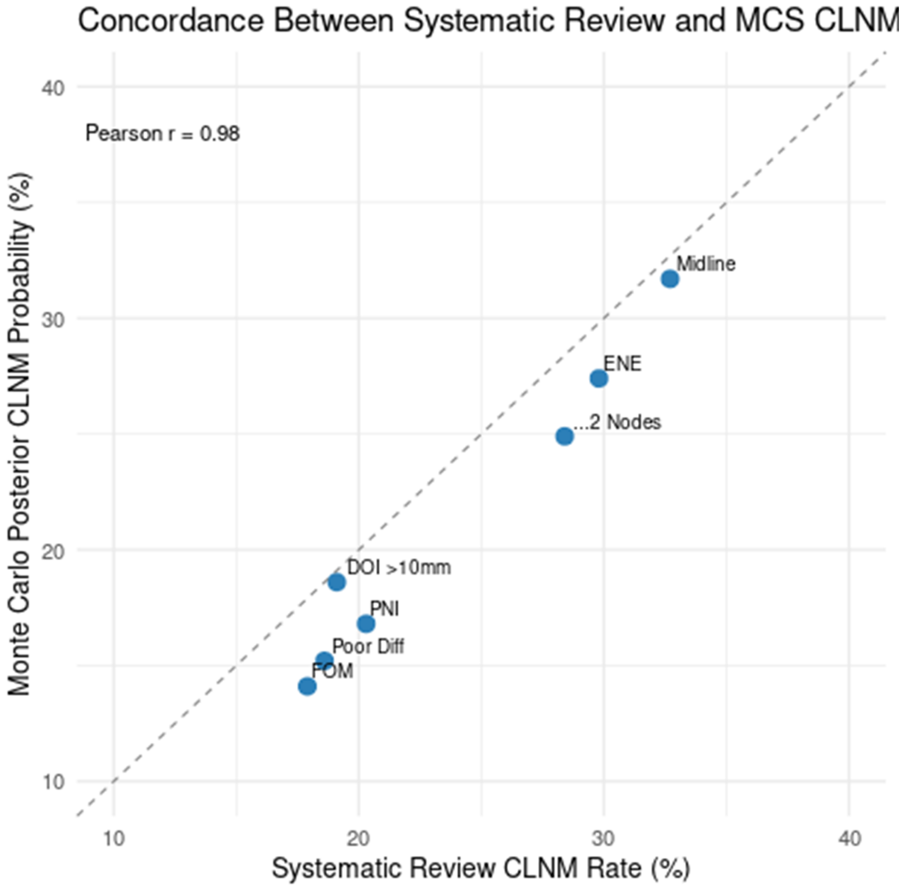

A strong linear correlation (Pearson r = 0.98) was observed, validating the calibration and robustness of the Bayesian model. Each point represents a risk factor, and its position along the identity line indicates the agreement between observed and modeled CLNM probabilities.
